# Genetic Analysis Workshop 15: simulation of a complex genetic model for rheumatoid arthritis in nuclear families including a dense SNP map with linkage disequilibrium between marker loci and trait loci

**DOI:** 10.1186/1753-6561-1-s1-s4

**Published:** 2007-12-18

**Authors:** Michael B Miller, Gregg R Lind, Na Li, Soon-Young Jang

**Affiliations:** 1 Division of Epidemiology and Community Health, School of Public Health, and Institute of Human Genetics, University of Minnesota, 1300 S Second Street, Suite 300, Minneapolis, Minnesota 55454, USA; 2 Division of Epidemiology and Community Health, School of Public Health, University of Minnesota, 1300 South 2nd Street, Suite 300, Minneapolis, Minnesota 55454, USA; 3 Division of Biostatistics, School of Public Health, University of Minnesota, Minneapolis, Minnesota 55454, USA

## Abstract

Data for Problem 3 of the Genetic Analysis Workshop 15 were generated by computer simulation in an attempt to mimic some of the genetic and epidemiological features of rheumatoid arthritis (RA) such as its population prevalence, sex ratio, risk to siblings of affected individuals, association with cigarette smoking, the strong effect of genotype in the HLA region and other genetic effects. A complex genetic model including epistasis and genotype-by-environment interaction was applied to a population of 1.9 million nuclear families of size four from which we selected 1500 families with both offspring affected and 2000 unrelated, unaffected individuals all of whose first-degree relatives were unaffected. This process was repeated to produce 100 replicate data sets. In addition, we generated marker data for 22 autosomes consisting of a genome-wide set of 730 simulated STRP markers, 9187 SNP markers and an additional 17,820 SNP markers on chromosome 6. Appropriate linkage disequilibrium between markers and between trait loci and markers was modelled using HapMap Phase 1 data . The code base for this project was written primarily in the Octave programming language, but it is being ported to the R language and developed into a larger project for general genetic simulation called GenetSim . All of the source code that was used to generate the GAW 15 Problem 3 data is freely available for download at .

## Background

The plan for this data simulation was to mimic the epidemiology and familial pattern of rheumatoid arthritis (RA), a complex genetic disease with several loci contributing to susceptibility. We took into account the following facts about RA epidemiology when choosing parameters for the simulation. The lifetime morbid risk of RA is roughly 1%, but risk in women is about triple the risk in men. Severity of illness is variable in RA but age of onset is only weakly related to disease severity and mortality is only slightly increased in RA. The risk to siblings of affected people is about five to ten times the risk in the general population, which suggests some genetic influence, and we know that the contribution of variation at *HLA-DRB1 *(OMIM: 142857) on chromosome 6 is extraordinarily strong and well established. In terms of environmental contributors to risk, we know that cigarette smoking is positively associated with RA. Two quantitative traits measured from serum, anti-cyclic citrullinated peptide (anti-CCP) and immunoglobulin M (IgM), are often elevated in RA. Much more is known about RA, but we focused our simulation on the features of the illness and its covariates listed here. Anything else that was known about RA genetics was ignored and we created a genetic model that we hoped would provide interesting data for Genetic Analysis Workshop participants.

In the simulation of trait loci and genetic marker data for linkage studies, it is very difficult to provide an appropriate model of linkage disequilibrium (LD) between loci, including both markers and trait loci. In many earlier GAW simulations, interest was primarily in linkage analysis and it was acceptable for all loci to be in linkage equilibrium. Interest today has shifted much more to association analyses using large numbers of single-nucleotide polymorphism (SNP) markers. For simulated genetic data to be useful, the simulation must include an appropriate LD model. Our approach was not to model LD explicitly but to use real SNP haplotype data from the HapMap project to provide appropriate local LD patterns. By recombining HapMap haplotypes in a realistic way, it is possible to produce large numbers of different chromosome-long haplotypes that retain the LD pattern of the original HapMap data over short intervals (i.e., <1 cM) but show much more haplotype diversity over longer intervals (e.g., 20 cM) than is observed in HapMap because of HapMap's limited sample size (120 haplotypes per ethnic group). In our simulation, every allele, whether marker allele or trait-locus allele, has an ancestral origin on one of the 120 CEPH European (CEU) HapMap haplotypes and this ancestry is what determines local LD patterns.

## Methods

### Errors in the Problem 3 data

After GAW15 participant manuscripts had been submitted for publication we discovered that there were several small errors in the Problem 3 simulation, none of which were noticed by participants. These errors were mostly trivial mistakes in the positions of some markers and trait loci and in the heterozygosities of the STRP markers. We present the simulation parameters below as they were presented to the GAW participants, but we note with asterisks any values that turned out to be incorrect and we then present the correct values. The most important error was in the positioning of Locus D relative to Locus DR/C: We intended for there to be only a 5 cM distance between those two loci on the sex-averaged map, but due to an error in the code for generating recombination in that region, the interval was roughly doubled to about 10 cM. This error should have almost no effect on association analyses because LD patterns were not affected. Marker order was always presented correctly. Another way to conceptualize the problem is that some of the map locations were presented incorrectly but the data were otherwise correct. All of our source code and other information about the Problem3 data is freely available at  so that the simulation process can be studied in detail by any interested reader. None of the software used in this project is proprietary or unavailable.

### Overview of the simulation process

We used a two-stage simulation process in which the first stage consisted of generating a large population of families with affection status, and the second stage consisted of generating full data for a much smaller number of families, selected based on affection status. Both stages were repeated 100 times to produce 100 replicate data sets.

#### Stage 1

We generated a large population of 1.8 million nuclear families, each consisting of two parents and two offspring, with RA affection status determined by the complex genetic/environmental model described below. At this stage of the analysis we generated for every family only the variables that were needed to determine affection status. These variables included age, sex, smoking status, IgM, and the trait loci described below. We did not determine patterns of recombination at this stage except in the regions between pairs of trait loci that were on the same chromosomes nor did we generate any marker data. For trait loci, we determined both the alleles and the HapMap haplotypes from which those alleles were derived. It was necessary to retain that haplotype data so that we could generate markers in LD with the trait loci in the second stage of the simulation process.

#### Stage 2

From the large population of families generated in Stage 1, we retained a random sample of 1500 families with an affected sibling pair (ASP) and we retained a random sample of 2000 control families in which none of the four members were affected. We then generated for the selected families all data, including marker data, that had not been generated in the first stage of the simulation. Use of this tactic required that the second stage of simulation was conditional on the results of the first stage. All 22 human autosomes were simulated. Information about the marker data is given below.

#### Data files

We present data on phenotypes and marker genotypes for all members of the 1500 ASP families and on one randomly selected offspring from each of the 2000 unaffected control families (i.e., there are 2000 unrelated control subjects per replicate, and no control subject had a first-degree relative with RA). Those data were shared with all GAW15 participants who requested Problem3 data, and for those participants who requested the answers, we also made available all trait-locus genotypes and latent traits that were used to generate observed quantitative traits.

### Major gene effects

**Figure 1:**
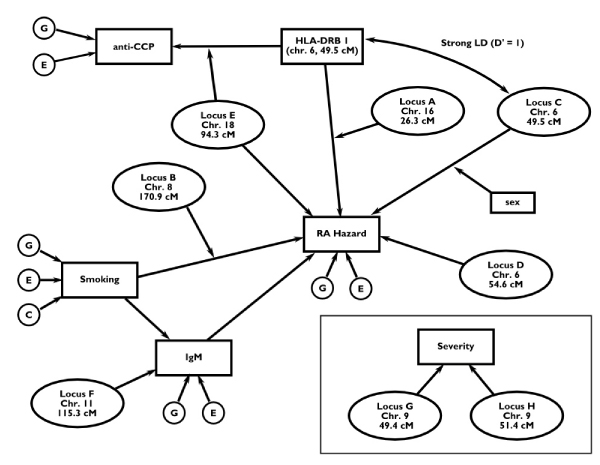
**The model for the GAW15 Problem 3 genetic simulation**. Genetic loci are represented as ovals, normally-distributed polygenic/environmental variables are represented as circles (G, additive polygenic; C, common family environment; E, non-shared environment) and observed variables are represented as rectangles. The RA hazard is a continuous variable that is dichotomized into affected/unaffected before it is observed, and severity is polytomized into five levels before it is observed. Arrows indicate where effects of variables are manifested. For example, HLA-DRB1 affects both anti-CCP levels and RA hazard, but the strength of its effect on anti-CCP is controlled by Locus E genotype and the effect of HLA-DRB1 on RA hazard is controlled by Locus A genotype.  The incorrect cM locations for Loci D and H are given, see the note on Table 1.

Table 1 summarizes the trait locus positions and provides a brief description of the effects of all nine major genes. Much of this information is also displayed in Figure 1. More detailed explanations of these effects follow immediately below.

**Table 1:** Effects of major genes

Locus	Chr	cM	Trait locus effect
DR	6	49.4556	Affects directly the risk of RA
A	16	26.2879	Controls effect of DR on RA risk
B	8	170.9087	Controls effect of smoking on RA risk
C	6	49.4556	0 cM from DR, increases RA risk only in women
D	6	54.5717^a^	5.12^a^ cM from DR, rare allele increases RA risk 5-fold
E	18	94.2729	Controls effect of DR on anti-CCP and increases RA risk
F	11	115.2864	QTL for IgM
G	9	49.3955	2 cM from Locus H, is 25% QTL for severity
H	9	51.4134^a^	2 cM from Locus G, is 25% QTL for severity

^a^These incorrect positions were reported to GAW participants. The correct positions are for Locus D: 59.4756 cM, 10.02 cM from Locus DR/C, and for Locus H: 51.2555 cM which is 1.86 cM from Locus G.

#### Hazard and risk

The model uses a constant hazard function to determine risk of RA. We planned at first to determine age at onset according to this exponential survival model, but it turned out that age of onset was then too strongly linked to some loci. We then retained the hazard approach but gave every individual the same risk period and the same "base hazard" (exponentiated intercept term). Therefore, multiplying hazard by some value is equivalent to multiplying risk by that value and the terms "risk" and "hazard" are used somewhat interchangeably below. Once hazard was known for a subject, we used the hazard to determine the mean of an exponential random variable. If this variable was less than a fixed threshold value (i.e., within the risk period), the subject was affected. The values of the base hazard and threshold are arbitrary, but they jointly determine population prevalence. Also see the section "RA affection status" under "Modeling phenotypes" below.

#### HLA-DR and Locus C

We imitated the HLA-DRB1 locus and its effects somewhat but not in great detail. We will refer to our simulated locus as "DR". Our model includes three DR alleles. DR is in strong LD (multi-allelic D' = 1.0) and complete linkage (0 recombination fraction) with Locus C. DR effects are independent of locus C effects, but DR effects are epistatically controlled by Locus A. In females only, each C allele increases risk by a factor of 2.1 (female risk of RA is multiplied by 2.1 for the Cc genotype and by 4.41 for the CC genotype). Females with no C alleles (cc) have no increased risk. The allele frequency for C is 0.5. DR/C haplotypes are shown in Table 2.

**Table 2:** DR/C haplotype frequencies (showing LD)

	C	c	
DR4	0.2500	0.0000	0.25
DR1	0.1000	0.0000	0.1
DRx	0.1500	0.5000	0.65
	0.5000	0.5000	1

Multiallelic: D = 0.15, D' = 1.0.

#### HLA-DR and Locus A

Locus A affects the impact of HLA-DR types in a dominant fashion. Individuals with Aa or AA genotypes have their hazard multiplied by a value that is determined by their DR type according to the "Risk Multipliers" (Table 3). A value of 1 indicates no change in risk. The allele frequency for A is 0.3 (thus the "aa" genotype has frequency 49% and "A_" has frequency 51%).

**Table 3:** Average DR risk for RA (across Locus A genotypes)

	DRX	DR1	DR4
DRX	1	1	5
DR1	1	1.5	6
DR4	5	6	30
			
Average DR risk multipliers			
DRX	0.8	1	1
DR1	1	6	6
DR4	1	6	2
			
DR risk multipliers for aa genotype: 49% frequency			
DRX	1.11359	1	5
DR1	1	0.42254	1.69014
DR4	5	1.69014	19.86755
			
DR risk multipliers for Aa or AA genotype: 51% frequency			
DRX	0.89087	1	5
DR1	1	2.53521	10.14085
DR4	5	10.14085	39.7351

#### Locus B

In smokers only, Bb or BB genotype multiplies RA risk by 1.5. This has the effect that smokers have no directly increased risk if their genotype is bb, but they still have some indirectly increased risk through the effect of smoking on IgM. The allele frequency for allele B is 0.35.

#### Locus D

Locus D has a direct effect on RA risk but a low allele frequency. Each D allele multiplies hazard by 5. The D allele frequency is only 0.0083 (exactly 1/120; so DD homozygotes are very rare).

#### Locus E

This locus has a strong direct effect on RA hazard, multiplying by 2.2 for each E allele (2.2 for Ee and 4.84 for EE). Locus E also affects anti-CCP by controlling which DR genotypes place a subject in the "high-mean" anti-CCP group (see also the anti-CCP section below). For DR4 homozygotes only, having one or more E alleles puts them in the group with a high mean anti-CCP level. Thus, the high-mean group for anti-CCP consists entirely of DR4 homozygotes with either Ee or EE genotypes. The frequency of the E allele is 0.25.

#### Locus F

An additive effect of locus F causes 30% of the variance in IgM. Mean values of IgM are proportional to number of F alleles. The frequency of the F allele is 0.5.

#### Loci G and H

These two diallelic loci have allele frequencies of 0.1 and 0.2, respectively, and each contributes an additive genetic effect that accounts for 25% of the variance of latent severity (a total of 50% jointly). These loci are about 2 cM apart on chromosome 9, but they are not in LD. Thresholds on latent severity are used to produce observed severity.

### Modeling phenotypes

#### Age

Ages for pairs of siblings were drawn from a bivariate normal distribution having parameters similar to pairs of affected siblings in real RA data we were given (rho = 0.855, SD = 11.51, mean = 54.60), but pairs were retained only if both ages were between 18 and 87. The mother's age at the birth of the oldest sibling was uniformly distributed between 20 and 30 years and the father's age was equal to the mother's age plus a triangular random variable with a range from -1 to 5 and a mean of 2. This kept all ages reasonable and within acceptable ranges. The age reported for deceased individuals is the age they would have been at ascertainment of their oldest child, if they had lived. Age at death is also reported for deceased parents.

#### Sex

The sex of offspring was determined by age from published census data on sex ratio by age.

#### Death and age of death

All offspring are living. The variable "dead" has value 1 for parents who were deceased at the time of ascertainment, and value 0 for parents who were living. A parent was determined to be dead based on 2002 Centers for Disease Control (CDC) mortality statistics for 10-year age classes. We applied a constant hazard within all but the oldest age group and started at the age of the parent when the youngest child was born. In the oldest age group (85 to 100 years), the density for age at death had a linear form with the mode at 85 and zero density at 100, limiting longevity to age 100. We present data for dead parents as if they were alive. Age at death is provided. RA has a small mortality effect. The age of death for affected parents is on average 2 years (symmetrical triangular distribution with endpoints 0, 4) earlier than expected.

#### RA affection status

Affection was determined by taking a fixed threshold on an exponential random variable (values below threshold were affected). The mean of the exponential random variable (reciprocal of the hazard) was determined by multiplication of risk factors. More precisely, the log-hazard was modeled as a linear function of risk factors and the individual exponential mean was 1/exp(log-hazard). This is a proportional hazards model with constant hazard and fixed follow-up time. Mortality and age were ignored in determining affection status. Variables and parameters that determined hazard are described below.

#### Smoking status

This was based on an age-dependent threshold model. A normal (0,1) random variable was generated for every subject such that variance was due to additive polygenic (50%), shared environmental (40%), and non-shared environmental (10%) influences. These numbers were based on results of a published twin study. Parents were genetically independent. Thresholds were determined by age according to CDC data so that individuals whose normal value exceeded a threshold were considered to be lifetime smokers at a probability appropriate for their age.

#### IgM

We generated a latent IgM value from a normal mixture with means determined by Locus F. Variance in latent IgM is caused by smoking status (24%), additive effect of Locus F (30%), and a residual (46%), with the residual variance being divided between additive polygenic (60%), and non-shared environmental (40%) components. The IgM latent variable was transformed monotonically to fit the distribution of IgM in real RA data.

#### Anti-CCP

Locus E and HLA-DR genotype jointly created 10.3% of the variance in anti-CCP as described in the "Locus E" section above. The remaining variance was caused by additive polygenic (60%), and non-shared environmental (40%) components. The anti-CCP latent variable was rank rescaled using the observed distribution of values in the RA reference data to create the final anti-CCP values.

#### Severity

Severity was determined by two diallelic loci (G and H, allele frequencies of 0.1 and 0.2, respectively) with additive effects. Each of the loci accounts for 25% of the total variance. The remaining variance (50%) is due to an individual random environment effect. There are 5 severity classes, each containing 20% of the affected persons.

#### Age of Onset

The age of onset (for affected offspring) was created from an "onset" latent variable that equally weighed the hazard, latent severity, and an independent random variate. This variable was converted to ranks and used with real RA data to derive a "proportion of life affected," which multiplied by the ascertainment age, yielded the age of onset.

#### Residual effect

There is a residual effect on the log-hazard for RA that is composed of shared environment effect (85% of variance) and a non-shared environment effect (15%). The shared environment effect is a constant multiple of a Bernoulli-distributed random variable and it is shared by all members of a family in 30% of families. The non-shared environment effect was normally distributed. The convolution (sum of the two variables) was a normal mixture with a standard deviation of 2.079.

### Summary of key covariate effects

#### Smoking

Smoking affects RA risk both directly with Locus B moderation and through its effect on IgM.

#### Age

Age affects RA risk through its affect on smoking and through its effect on the sex ratio. Age affects mortality, but only in the parents, and we report affection status regardless of mortality.

#### Sex

Nearly all of the sex effect in RA risk comes from Locus C, but the sex ratio in the general population, which was based on CDC data, also has an effect in the offspring generation.

### Marker data

We present markers on 22 autosomes that were designed to be like real human autosomes in terms of genetic and physical map lengths, but we did not generate data for sex chromosomes. The markers were presented in three sets:

1. A set of 730 microsatellite markers, fairly evenly spaced on chromosomes with an average inter-marker distance of about 5 cM and with heterozygosities always exceeding 0.70 (due to a programming error, microsatellites had lower heterozygosities than this).

2. A set of 9187 SNPs distributed on the genome to mimic an Affymetrix 10 K SNP chip set but without monomorphic SNPs. Because these SNPs were derived from 120 HapMap haplotypes, all SNP allele frequencies were integer multiples of 1/120 (0.0083) and the lowest frequency was precisely 1/120.

3. A very dense map of 17,820 SNPs on chromosome 6 (an average inter-marker spacing of 9586 bp, which corresponds roughly to the density one would expect from a genome-wide 300 K SNP set). The chromosome 6 dense map includes 210 of the markers from the 10 K SNP map (they are easily identifiable because they have the same names in both sets).

#### Haplotype generation

Offspring haplotypes were generated by dropping genes from parental haplotypes in the usual Mendelian fashion and assuming a Haldane model (no interference). Parental haplotypes were derived by recombining the 120 HapMap CEU haplotypes under the assumption of Hardy-Weinberg conditions and assuming 30 generations of random mating from a large population where the 120 HapMap haplotypes initially had equal frequency. This was done by generating a Poisson-distributed random variable with a mean of 30 times the length of the chromosome in Morgans and then distributing uniformly along the chromosome the Poisson-distributed number of recombination points. To every interval between recombination points, one of the 120 HapMap haplotypes is then assigned at random. Any regions that held a trait locus had to be assigned in Stage 2 of the simulation whichever HapMap haplotype was assigned to the trait locus in Stage 1.

#### Special features of the simulated marker data

We provided more information in the simulated data than one would ordinarily have in real data. These special features of the data include the following:

##### 1. No missing data

Marker data are usually missing on some family members, especially those who died before the family was ascertained. We provide marker data on all family members. Researchers who would like their data to be more realistic can delete marker information from deceased individuals. By supplying data that would normally be missing, we provide more opportunity to test effects of missingness, etc.

##### 2. No errors

We did not model any errors in the data simulation. In real data there are typically some errors in genotyping and sometimes there are sample mixups. By not modeling any errors, we make it possible for the analyst to simulate his own errors and test the effect of genotyping error on other aspects of a genetic analysis. We also added no errors to phenotypes.

##### 3. Allele ordering reveals phase

The allele inherited from the father is always presented on the left side within every genotype. This allows researchers to determine haplotypes for all subjects and to determine their parental origin. In real data, it is usually not possible to know haplotypes or their origin, but new methods have made molecular haplotyping possible and it is currently being used. So, in real data we can sometimes know haplotypes, but the parental origins of those haplotypes still must be inferred.

## Results

### Epidemiological data from Stage 1 of the simulation

We analyzed the data from a randomly selected general population of 1.8 million families in our Stage 1 simulation to see that our epidemiological parameters had the correct values. The results for 1.8 million sibling pairs (3.6 million subjects) generated using the model we developed are shown in Table 4.

**Table 4:** Epidemiological parameters estimated from 1.8 million simulated sibling pairs

Parameter	
RA lifetime prevalence	1.07%
F:M sex ratio in affecteds	3.07
Sibling relative risk	9.03
Number of ASPs	1856 (of 1.8 million sibling pairs)

The sibling relative risk is the lifetime prevalence in siblings of affected individuals (proband-wise concordance) divided by the lifetime prevalence in our simulated general population. The numbers in Table 4 above are similar to what one would see in real RA epidemiological data. These numbers apply only to the offspring generation, and not the parent generation of our simulated data.

### Genetic analysis

After generating the Problem 3 data we undertook a series of genetic analyses to check the data for flaws. We were satisfied, despite the very pronounced affect of the HLA locus on RA. This effect may be too large, but it does reflect the actual effect found in real RA data. The results of these analyses are available at .

## Discussion

The use of software developed in an interpreted language such as Octave [1] or R [2] can be used to produce very sophisticated genetic data sets. Modern computers are fast and powerful enough to compensate for the slower speed of such software and the development time is speeded up greatly when functions developed in the interpreted language can be used. Slower functions can be written in C++ and compiled to work within the Octave or R environment, thus speeding up the simulation.

The data contained a few small errors, but these probably did not have important effects on GAW15 participant research. We provide information on our web page at  about the Problem 3 data, including the full source code used to produce it, and we will continue to update that web page if more information becomes available about the data (e.g., answers to user questions or previously undocumented features). Because we stored the seeds used to generate the Problem 3 data, it is theoretically possible to reproduce it in its entirety. We have done this but it does require a computer with considerable amounts of RAM (maybe 10 GB). We are adding code on our web page that will allow users to generate smaller data sets under the same model and thus circumvent the memory requirement.

## Competing interests

The author(s) declare that they have no competing interests.
